# Low concentrations of saracatinib promote definitive endoderm differentiation through inhibition of FAK-YAP signaling axis

**DOI:** 10.1186/s12964-024-01679-7

**Published:** 2024-05-30

**Authors:** Ruiyang Ma, Huanjing Bi, Ying Wang, Jingwen Wang, Jiangwei Zhang, Xiaoyang Yu, Zuhan Chen, Jiale Wang, Cuinan Lu, Jin Zheng, Yang Li, Xiaoming Ding

**Affiliations:** Department of Renal Transplantation, Hospital of Nephrology, The First Affiliated Hospital of Xi’an Jiaotong University, 277 Yanta Western Rd, Xi’an, Shaanxi Province 710061 China

**Keywords:** Definitive endoderm, Focal adhesion kinase, Human pluripotent stem cells, Saracatinib, Yes-associated protein

## Abstract

**Abstract:**

Optimizing the efficiency of definitive endoderm (DE) differentiation is necessary for the generation of diverse organ-like structures. In this study, we used the small molecule inhibitor saracatinib (SAR) to enhance DE differentiation of human embryonic stem cells and induced pluripotent stem cells. SAR significantly improved DE differentiation efficiency at low concentrations. The interaction between SAR and Focal Adhesion Kinase (FAK) was explored through RNA-seq and molecular docking simulations, which further supported the inhibition of DE differentiation by p-FAK overexpression in SAR-treated cells. In addition, we found that SAR inhibited the nuclear translocation of Yes-associated protein (YAP), a downstream effector of FAK, which promoted DE differentiation. Moreover, the addition of SAR enabled a significant reduction in activin A (AA) from 50 to 10 ng/mL without compromising DE differentiation efficiency. For induction of the pancreatic lineage, 10 ng/ml AA combined with SAR at the DE differentiation stage yielded a comparative number of PDX1^+^/NKX6.1^+^ pancreatic progenitor cells to those obtained by 50 ng/ml AA treatment. Our study highlights SAR as a potential modulator that facilitates the cost-effective generation of DE cells and provides insight into the orchestration of cell fate determination.

**Graphical Abstract:**

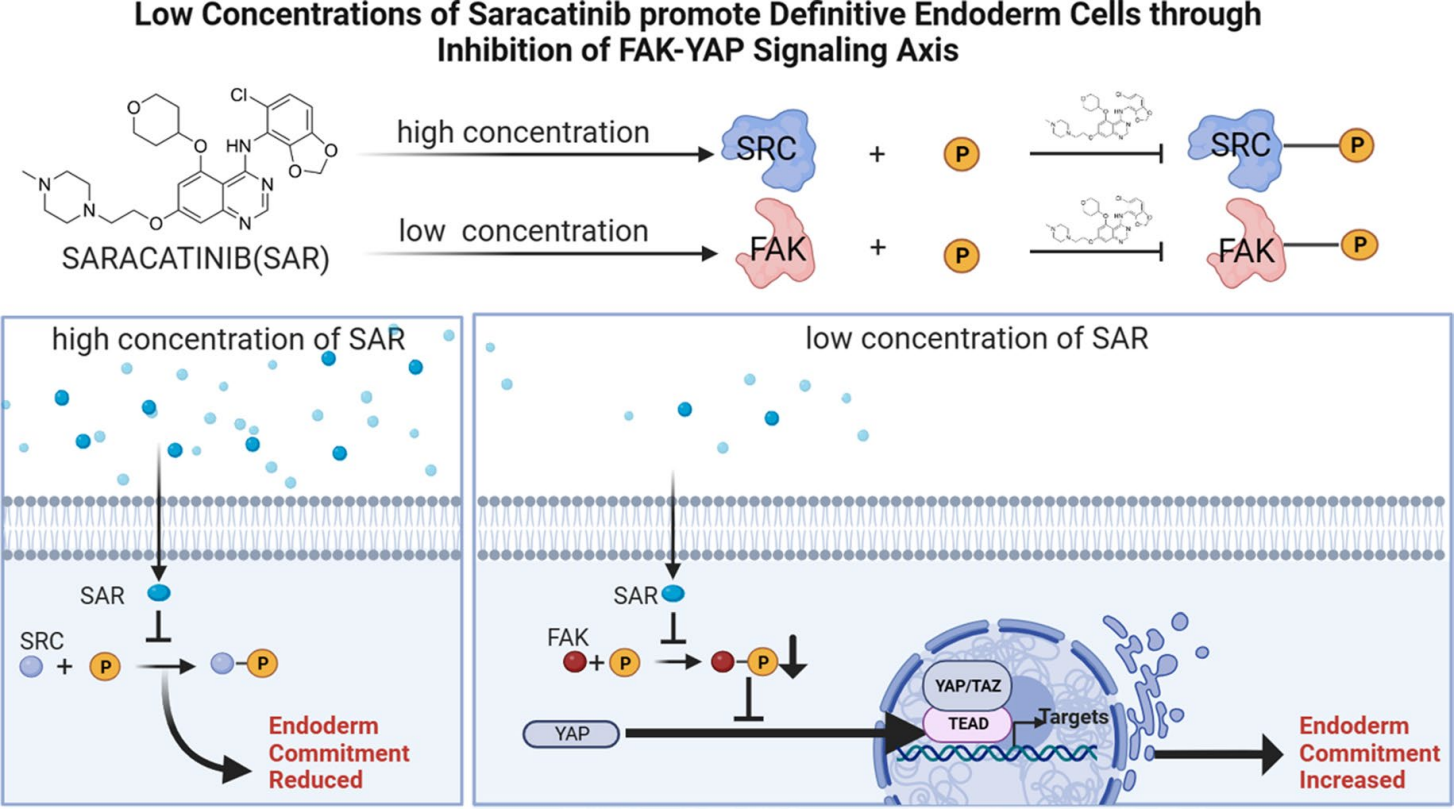

**Supplementary Information:**

The online version contains supplementary material available at 10.1186/s12964-024-01679-7.

## Introduction

Pluripotent stem cells (PSCs), including embryonic stem cells (ESCs) and induced pluripotent stem cells (iPSCs) [1], have the potential to differentiate into all three embryonic germ layers: ectoderm, mesoderm, and definitive endoderm (DE). DE gives rise to the epithelial lining of the respiratory and digestive tracts as well as the thyroid, thymus, lungs, liver, and pancreas [2]. Thus, they have significant clinical value for regenerative medicine and drug development [3]. For example, the development of pancreatic organoids holds promise for the treatment of diabetes through pancreatic transplantation [4, 5]; however, there is significant potential for further improving the extent and efficiency of the DE differentiation process. Currently, the DE differentiation protocol primarily involves activin A (AA) and CHIR-99,021 [6]. AA is the most important and principal inducer of endodermal differentiation and is associated with the TGF-β pathway, which induces the expression of endodermal genes [2, 7]. Because of the high cost, large consumption, and challenges for protein stability associated with AA, the differentiation protocol is not economically viable, although attempts have been made to replace AA with small molecules [8]. Nevertheless, a recent study demonstrated that a high dose of AA (100 ng/ml) causes higher cell death during endoderm differentiation [9].

Saracatinib (SAR) is an orally available 5-, 7-substituted anilinoquinazoline compound that is known for its anti-invasive and antitumor properties [10]. Functioning as a dual-specific small molecule inhibitor, SAR targets the Src and Abl kinases by specifically interacting with the tyrosine kinase domain to inhibit their activity. SAR interacts with the ATP-binding site through a mechanism that is similar to ATP-competitive Src kinase inhibitors [11]. As a result, SAR significantly influences a variety of cellular functions, including motility, migration, adhesion, invasion, proliferation, differentiation, and survival [12]. Because of the high similarity among tyrosine kinase domains, the potential for off-target effects exists. Therefore, as a member of the nonreceptor tyrosine kinase family, targeting Src with the SAR inhibitor may result in off-target effects [13].

Focal adhesion kinase (FAK), like Src, is a nonreceptor tyrosine kinase and is associated with various processes, including the epithelial–mesenchymal transition (EMT) [14], differentiation, and tumor cell proliferation [15, 16]. FAK is a widely expressed cytoplasmic protein-tyrosine kinase that is activated by integrin ligation and clustering, growth factor stimulation, and G-protein-linked receptor activation [17–20]. The kinase domain of nonreceptor tyrosine kinases is highly homologous to the kinase domain of FAK and Src, which is the basis for our hypothesis that SAR exhibits off-target effects [21–23]. Yes-associated protein (YAP) is an important component of the Hippo signaling pathway [24]. It plays a central role in regulating organ size, cell proliferation, stem cell activity, and tumor development [25–27]. The cellular localization of YAP regulates its activity. Cytoplasmic YAP is recruited to the disruption complex, ubiquitinated, and degraded [28], whereas nuclear YAP induces gene transcription in association with the TEA domain transcription factor (TEAD) [29, 30]. FAK activation increases the nuclear localization of YAP, which in turn, triggers a cascade of downstream transcription factors regulated by YAP. FAK inhibitors can prevent the accumulation of YAP in the nucleus [31]. Currently, there is little data regarding the role of the FAK–YAP signaling axis in DE differentiation.

In this study, we examined the role of SAR in promoting the differentiation of ESCs and iPSCs into DE cells. The results represent the first demonstration that low concentrations of SAR promote DE differentiation. We also elucidated the mechanism underlying this differentiation-promoting effect. Using RNA-seq, we discovered that SAR directly interacts with FAK and inhibits its activity by phosphorylating the Y397 site of FAK. Deactivated FAK further suppresses the nuclear translocation of YAP to promote the differentiation of PSCs into DE.

## Materials and methods

### Cell culture and differentiation

Human hESC lines H1 and H9 were purchased from the WiCell Research Institute. The human fibroblast-derived iPSC line K0 was a kind gift from Dr. Xiong Guo [32]. The human pancreatic islet-derived iPSC line HECi001-A was a gift from HEC Pharm Co., Ltd [33]. These stem cells were maintained in PGM1 medium (Cellapy, CA1014500, Beijing, China) on Matrigel-coated cell culture plates at 37 °C in a 5% CO_2_ atmosphere. DE differentiation was performed as previously reported with slight modifications [34]. Briefly, the cells were dissociated using Accutase (StemCell Technologies, Vancouver, Canada) and seeded at a density of 1 × 10^6^ cells per well on Matrigel-coated 6-well plates. The differentiation medium was DMEM medium (BasalMedia, CAT#L110KJ, Shanghai, China) supplemented with B27 (BasalMedia, S440J7), Glutamax (BasalMedia, Cat# S240JV), 50 ng/mL AA (SinoBiological, Cat# 10,429-HNAH, Beijing, China), and 2.5 µM CHIR99021 (Meilunbio, Cat# MB5683, Dalian, China) and the cells were incubated for 24 h. Subsequently, CHIR99021 was removed from the medium for the following two days. Pancreatic progenitor cell differentiation was performed using a stepwise protocol containing 4 stages as previously described [35]. At Stage 2 (day 4–5), the media consisted of DMEM supplemented with 1 mg/mL BSA, ITS (BasalMedia, S450J7), Glutamax, 0.25 mM Vitamin C (Sigma-Aldrich, A4544, St. Louis, Missouri, USA), and 50 ng/mL keratinocyte growth factor (KGF) (SinoBiological, Cat: 10,210-H07E). At Stage 3 (day 6–7), the cells were cultured in DMEM supplemented with 1 mg/mL BSA, ITS, Glutamax, 50 ng/mL KGF, 0.25 mM Vitamin C, 2 µM retinoic acid (RA, Sigma-Aldrich, R2625), 0.2µM TPPB (TargetMol, T17148, Boston, Massachusetts, USA), 200 nM LDN193189 (MCE, HY-12,071, NJ, USA), and 0.25 µM Sant1 (Targetmol, Cat T2450). At Stage 4 (day 8–12), the differentiation media contained DMEM medium, 1 mg/mL BSA, ITS, Glutamax, 50 ng/mL KGF, 0.25 mM Vitamin C, 100 nM RA, 0.2 µM TPPB, 0.25 µM Sant1, and 200 nM LDN193189. The small molecules used in this study included SAR (TargetMol, T6078) and VP (TargetMol, T3112). All the differentiation medium was changed on a daily basis.

### Western blot analysis

Cell extracts were prepared using RIPA Lysis Buffer (Beyotime, cat# P0013B, Shanghai, China) supplemented with ProtLytic Protease and Phosphatase Inhibitor Cocktail (New Cell Molecular Biotech). Nuclear proteins were extracted using the Nuclear and Cytoplasmic Protein Extraction Kit (Beyotime, cat# P0027). Protein concentration was measured using a BCA Protein Assay Kit (CWBIO, CAT#CW0014, Taizhou, China). Protein samples (10 µg) were loaded and separated by electrophoresis on 10% SDS-polyacrylamide gels and subsequently transferred to polyvinylidene difluoride membranes (Bio-Rad, Hercules, California USA). The membranes were incubated overnight at 4 °C with primary antibodies, including anti-phospho-Src-Y419 antibody (Abclonal, cat# AP1027, 1:1,000, Wuhan, China), anti-Src antibody (Abclonal, cat# A19119, 1:1,000), anti-phospho-FAK-Y397 antibody (Abclonal, cat# AP1447, 1:1,000), anti-FAK antibody (Abclonal, cat# A11131, 1:1,000), anti-FOXA2 antibody (Abclonal, cat# A19053, 1:1,000), anti-SOX17 antibody(Abclonal, cat# A18858, 1:1,000), anti-DYKDDDDK Tag antibody (CST, cat# 14,793, 1:4,000, Danvers, Massachusetts, USA), anti-phospho-FAK-Y925 antibody (Abclonal, cat# AP1098, 1:1,000), anti-phospho-FAK-Y576/577 antibody (Abclonal, cat# AP0536, 1:1,000), and anti-YAP1 antibody (Abclonal, cat# A19134, 1:1,000). Next, the membranes were washed and incubated with HRP-conjugated goat anti-rabbit IgG heavy chain (Abclonal, cat# AS063, 1:5,000) or HRP-conjugated goat anti-mouse IgG heavy chain (Abclonal, cat# AS064, 1:5,000) at room temperature for 1 h. For normalization, GAPDH antibodies (Abclonal, cat# AC002, 1:5,000) were used as an internal reference for total cellular protein, and Lamin B1 (Abclonal, cat# A11495, 1:2000) was used as the internal reference for nuclear proteins. Signal detection was done using an enhanced chemiluminescence western blotting detection kit (Bio-Rad, 1,705,061). The optical density of each band was quantified using ImageJ software.

### RNA preparation and qPCR

Total RNA was isolated using the SteadyPure Universal RNA Extraction Kit (ACCURATE BIOLOGY, Cat#AG21022, Changsha, China). Total RNA (0.5 µg) was subjected to reverse transcription to synthesize complementary DNA using the Evo M-MLV RT Kit with gDNA Clean for qPCR (ACCURATE BIOLOGY, Cat#AG11705). qPCR was performed using 2×SYBR Green qPCR Master Mix (ACCURATE BIOLOGY, Cat# AG11702) and a Bio-Rad CFX96 RT-PCR System. Ribosomal protein lateral stalk subunit P0 (RPLP0) was used as the internal reference gene for qPCR, and the 2^−ΔΔCt^ method was used to calculate relative expression. Detailed information regarding the primer sequences is listed in Supplementary Table 1.

### Immunofluorescent staining

Cells were cultured on tissue culture plates and fixed with 4% paraformaldehyde for 15 min at room temperature, permeabilized with 0.5% Triton X-100 in PBS for 15 min at room temperature, and blocked in 1% BSA for 30 min at room temperature. Primary antibodies prepared and diluted in primary antibody dilution solution were incubated with the cells overnight at 4 °C. The following day, the cells were thoroughly washed three times with PBS and incubated with secondary antibodies prepared in secondary antibody dilution solution for 45 min at room temperature. Finally, the cells were washed three times using PBS and subsequently stained with either Hoechst or PI for 10 min at room temperature. The primary antibodies included mouse anti-SOX17 (Santa Cruz, sc-130,295, 1:100, Dallas, Texas, USA), rabbit anti-FOXA2 (Abclonal, cat# A19053, 1:150), rabbit anti-YAP1 (Abclonal, cat# A19134, 1:500), rabbit anti-PDX1 (R&D SYSTEMS, Cat# AF2419, 1:200, Minneapolis, MN, USA), and goat anti-NKX6.1 (Abclonal, cat# A20419, 1:200). Immunoreactivity images were acquired and processed using either a confocal microscope (from Leica) or a conventional fluorescence microscope.

### Flow cytometry

For flow cytometric analysis, differentiated cells were dissociated using Trpzyme (BasalMedia, S342JV) for 2 min. The cells were fixed with 4% paraformaldehyde for 15 min at room temperature, washed once with FACS buffer (PBS with 1% BSA), centrifuged, and the supernatant was removed. Permeabilization was done by incubating with 0.5% Triton X-100 for 15 min at room temperature. Subsequently, blocking was done with 1% BSA for 30 min at room temperature. A 647-conjugated antibody targeting FOXA2 (Santa Cruz, cat# sc-377,033 AF647, 1:100) and a FITC-conjugated antibody against SOX17 (Santa Cruz, cat# sc-130,295 FITC, 1:100) were added and incubated for 30 min at room temperature. The resulting cell population was analyzed using a NovoCyte D3000 Advanteon flow cytometer (Agilent, CA, USA). The acquired FACS data were subsequently processed using FlowJo software.

### RNA sequencing and data analysis

Total RNA was isolated from the hESC line H1 using TRIzol reagent (Magen, R4801-01B, Guangzhou, China). The quality of the RNA samples was assessed based on the A260/A280 absorbance ratio, which was measured using a Nanodrop ND-2000 system from Thermo Scientific (USA). Initially, the mRNA was extracted from 1 µg of total RNA using oligo (dT) magnetic beads. Subsequently, total RNA was reverse-transcribed into cDNA fragments, which were then ligated with adapters to construct the paired-end library. Finally, the libraries were sequenced on an Illumina Novaseq 6,000 platform, which generated 150 bp paired-end reads. Following data cleaning, normalization, and annotation, differential expression analysis was performed using DESeq2 (http://bioconductor.org/packages/release/bioc/html/DESeq2.html). Genes with an absolute log_2_ fold change (| log_2_FC |) > 1 and an adjusted p-value < 0.05 were considered significantly differentially expressed. Differentially expressed genes between differentiated cells with or without 0.5 µM SAR treatment are shown in the Supplementary Table 2.

### Adenovirus-mediated overexpression of active FAK

The pADM-EF1a-FAK-Y397-3flag adenovirus was constructed and packaged by WZ Biosciences Inc. (Jinan, China) to facilitate the expression of a constitutively active FAK, in which the tyrosine residue at position 397 was substituted with aspartic acid. Empty adenovirus (Ad-NC) was used as a control. ESCs or iPSCs were seeded at a density of 1 × 10^6^ cells per well on Matrigel-coated 6-well plates. After 24 h, the cells were infected with Ad-FAK Y397D or empty adenovirus with a multiplicity of infection (MOI) of 50 in the presence of DE differentiation medium. After 24 h of infection, the medium was replaced with fresh differentiation medium. Infection efficiency was verified by western blot analysis at the end of DE differentiation.

### Molecular docking

Both SAR and FAK were subjected to molecular docking analysis using the CB-Dock2 platform (https://cadd.labshare.cn/cb-dock2/php/index.php). The 2D chemical structure of SAR was obtained from the PubChem database (https://pubchem.ncbi.nlm.nih.gov). Hydrogen atoms were added and free energy minimization was performed. In addition, the crystal structures of FAK (PDB ID: 6YQ1) were acquired from the RCSB PDB [36]. Hydrogen atoms were added and water molecules were removed. CB-Dock2 software was used to calculate the binding affinity energy (kcal/mol), resulting in the identification of the optimal docking model with the lowest energy. To visualize the specifics of ligand–receptor interactions, PyMol2 software (Free version) and Discovery Studio Visualizer (version: 2021) software were employed.

### Molecular dynamics simulation

Molecular dynamics simulations were conducted using Gromacs2022.3 software. Small molecule preprocessing involved the addition of the General Amber Force Field to small molecules using AmberTools22. Hydrogenation of small molecules and calculation of the RESP potential were done using Gaussian 16 W. The resulting potential data were incorporated into the topology file of the molecular dynamics system. The simulation conditions were set at a static temperature of 300 K and atmospheric pressure of 1 Bar. The Amber99sb-ildn force field was used with water molecules acting as the solvent (Tip3p water model). To neutralize the total charge of the simulation system, an appropriate number of Na^+^ ions was added. The simulation used the steepest descent method for energy minimization. Isothermal isovolumic ensemble equilibrium and isothermal isobaric ensemble equilibrium were carried out separately for 100,000 steps each, with a coupling constant of 0.1 ps and a duration of 100 ps. Then, a free molecular dynamics simulation was conducted, encompassing 5,000,000 steps, each with a step length of 2 fs for a total duration of 100 ns. Upon completion of the simulation, the software’s built-in tool was used to analyze the trajectory, and the root mean square deviation (RMSD) was calculated.

### Statistical analysis

All data are presented as the mean ± standard error of the mean. Statistical analysis was performed using a one-way ANOVA with Tukey’s post hoc test. A two-tailed unpaired Student’s t-test was performed for comparison between the two groups. Statistical significance was defined as *p* < 0.05, and the level of significance was denoted as follows: *p < 0.05, **p < 0.01, ***p < 0.001. Statistical analyses were conducted using GraphPad Prism software.

## Results

### Low concentrations of SAR promote DE differentiation

**Fig. 1:**
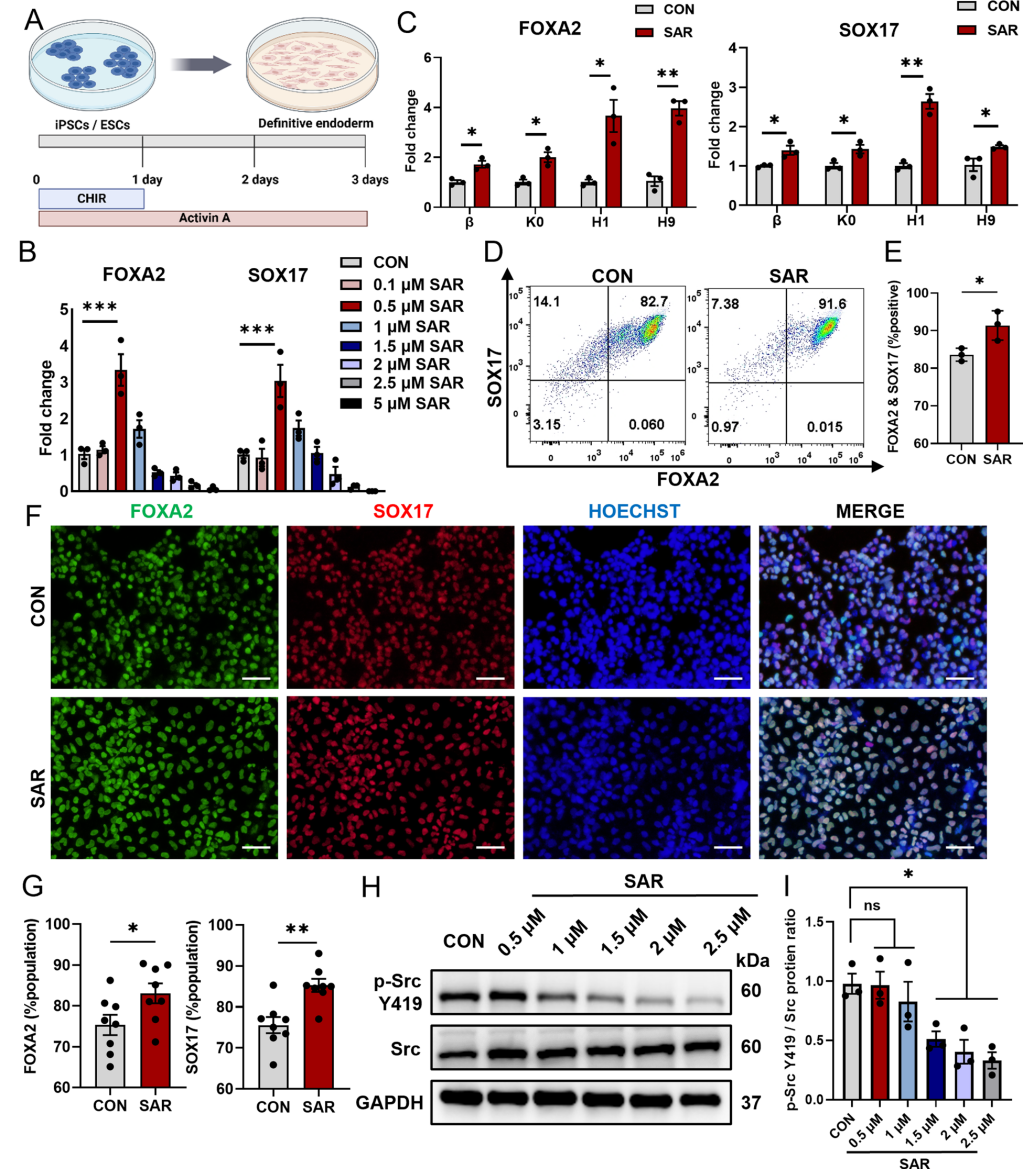
Low concentrations of SAR promote Definitive Endoderm (DE) differentiation. DE differentiation was performed using the previously reported protocol (**A**) for 3 days. After 3 days of differentiation, total RNAs were extracted and the expressions of FOXA2 and SOX17 in DE cells derived from the H1 cells were determined by qPCR (**B**). (**C**) QPCR results of FOXA2 and SOX17 in two different ESC lines (H1 and H9) and two iPSC lines (K0 and β) treated with 0.5 µM SAR. (**D**) Flow cytometric analysis of DE cells derived from hESC line H1 expressing FOXA2 and SOX17 with or without the treatment of 0.5 µM SAR. (**E**) Quantitative statistics of FOXA2^+^/SOX17^+^ cells corresponding to (**D**). (**F**) Immunofluorescent staining examined FOXA2^+^ (green) and SOX17^+^ (red) cells treated with 0.5 µM SAR differentiated from hESC line H1. The nucleus was counterstained with Hoechst 33,342. Quantitative statistics were shown in (**G**). (**H**) The p-Src Y419 and Src protein levels in DE cells treated with different concentrations of SAR were detected using western blotting. GAPDH was used as a loading control. (**I**) Quantitative statistics of p-Src Y419/Src corresponding to H. Data are presented as mean ± SEM (*n* = 3–8). *p < 0.05; **p < 0.01; ***p < 0.001; ns, not significant

We applied the most commonly used differentiation protocol based on AA [35] to generate DE cells (Fig. 1A). In our differentiation process supplemented with SAR, we observed notable and concentration-dependent alterations in cell morphology (Figure S1A). As the concentration of SAR increased and the duration of cultivation was extended, differentiating stem cells underwent a morphological transformation from a planar polygonal cell morphology to a multilayered semispherical cell morphology. The qPCR results indicated that low concentrations of SAR effectively promoted DE differentiation, with the highest efficiency observed at a concentration of 0.5 µM (Fig. 1B); however, SAR inhibited DE differentiation at concentrations higher than 1 µM, which was consistent with previous studies [37]. We optimized the duration of SAR treatment and found that the highest differentiation efficiency was achieved when SAR was used throughout the entire first stage of differentiation (Figure S1B and C). In order to verify the universality of this effect, we tested two human ESC lines (H1 and H9) and two iPSC lines (β and K0). We found that 0.5 µM SAR efficiently promoted DE differentiation in all four PSC lines (Fig. 1C). Furthermore, flow cytometry confirmed that a low concentration of SAR significantly enhanced DE differentiation efficiency (Fig. 1D and E). The promoting effect at low SAR concentrations was further supported by immunofluorescent staining (Fig. 1F and G). Because SAR was previously reported as an Src family inhibitor [38], we assessed phosphorylated Src levels by western blot analysis. SAR could not effectively inhibit the phosphorylation of Src at concentrations lower than 1 µM (Fig. 1H and I). Therefore, we speculated that DE differentiation, at low SAR concentrations, may not be facilitated via the inhibition of Src phosphorylation.

### SAR inhibits focal adhesion activation during DE differentiation

**Fig. 2:**
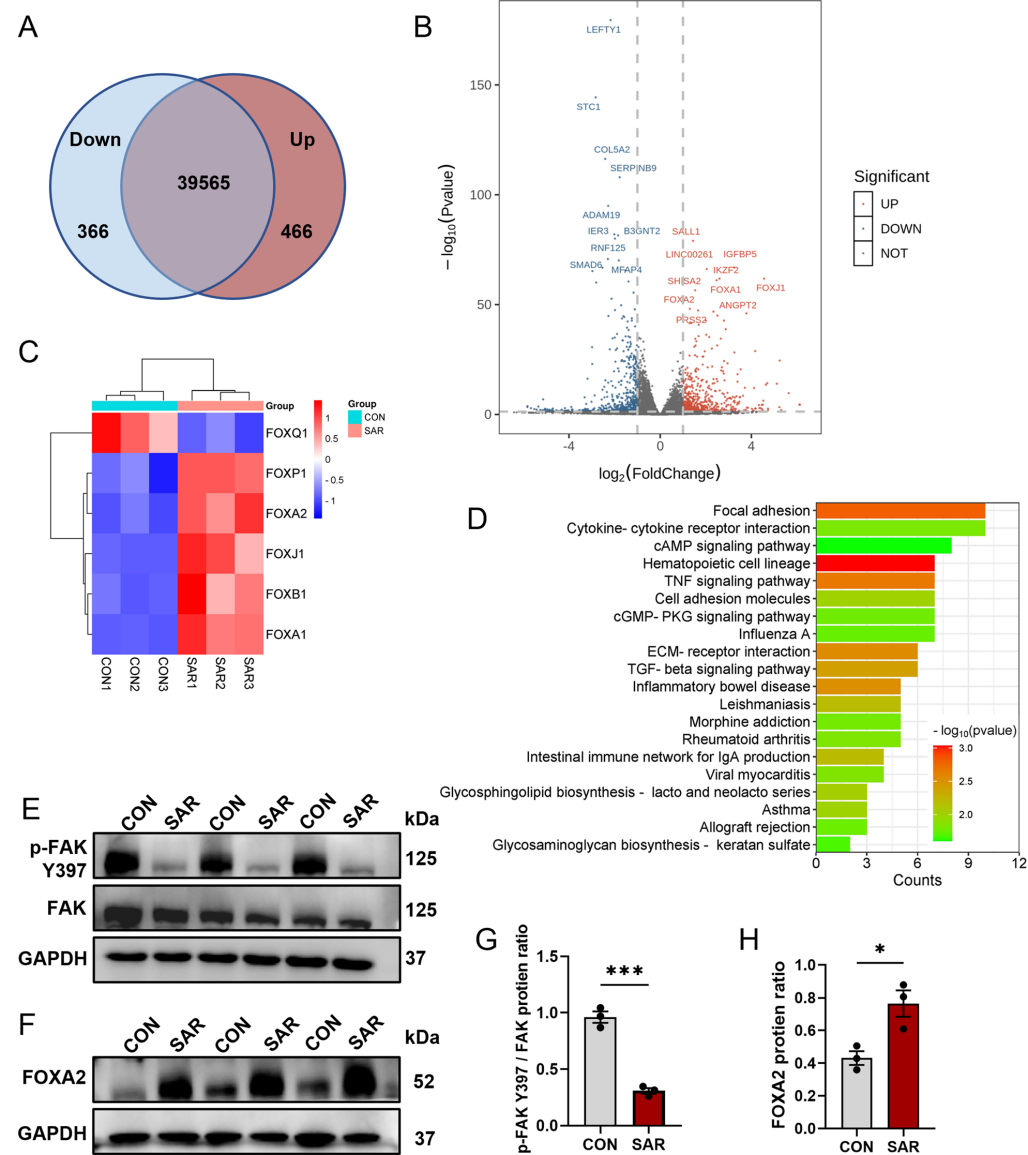
SAR inhibits focal adhesion activation during DE differentiation. RNA-seq was performed using differentiated cells from hESC line H1 with or without the addition of 0.5 µM SAR after 3 days of DE differentiation. (**A**) Venn diagram illustrating the number of upregulated and downregulated transcripts upon SAR treatment. (**B**) Volcano plot indicating the top ten upregulated and top ten downregulated genes based on -log10(P value) values. (**C**) Heatmap analysis of the FOX family factors among differentially expressed genes. (**D**) Top 20 KEGG enrichment terms of downregulated genes. After 3 days of differentiation, total protein was extracted from differentiated H1 cells to validate the results of RNA-seq. (**E**) P-FAK Y397 and total FAK in DE cells treated with 0.5 µM SAR or equal volume of DMSO were examined by western Blot. GAPDH was used as a loading control. (**F**) Western Blot results of FOXA2 and GAPDH in DE cells treated with 0.5 µM SAR or equal volume of DMSO. (**G**) Quantitative statistics of p-FAK Y397/FAK corresponding to (**E**). (**H**) Quantitative statistics of FOXA2 corresponding to (**F**). Data are presented as mean ± SEM (*n* = 3). *p < 0.05; ***p < 0.001

To determine the effect of 0.5 µM SAR on the DE differentiation of ESC H1 cells, we performed RNA sequencing (RNA-seq) analysis with differentiated cells treated with or without 0.5 µM SAR. The raw data were deposited in GEO database under submission number GSE251992. Our PCA results demonstrated a favorable outcome, with well-separated clustering and distinct patterns in the PC space (Figure S2A). We identified 40,397 transcripts, of which 466 were upregulated and 366 were downregulated (Fig. 2A and B). Interestingly, SAR treatment resulted in a significant upregulation of genes within the FOX family, including FOXA1, FOXA2, and FOXJ1 (Fig. 2C and S2B). Gene Ontology enrichment analysis of the differentially expressed genes indicated that SAR caused alterations in developmental processes, including anatomical structure morphogenesis, anatomical structure development, multicellular organism development, system development, and animal organ development (Figure S2C). To identify downregulated genes, we performed a KEGG pathway analysis, which indicated enrichment of genes associated with the focal adhesion pathway (Fig. 2D and S3). Numerous studies have shown a pivotal regulatory role for FAK in the focal adhesion pathway [16, 39]. The western blot results revealed substantial inhibition of Y397 phosphorylation of FAK following SAR treatment (Fig. 2E and G). Meanwhile, we also investigated other phosphorylation sites of FAK and found that Y576/577 were similarly inhibited, while there was no noticeable regulation of phosphorylation at the Y925 site. And SAR could not effectively inhibit the phosphorylation of Src (Figure S4A and B). In addition to assessing FAK phosphorylation, we confirmed increased FOXA2 protein levels following the addition of 0.5 µM SAR (Fig. 2F and H). These findings suggest a potential connection between SAR treatment and FAK signaling modulation.

### SAR directly targets FAK

**Fig. 3:**
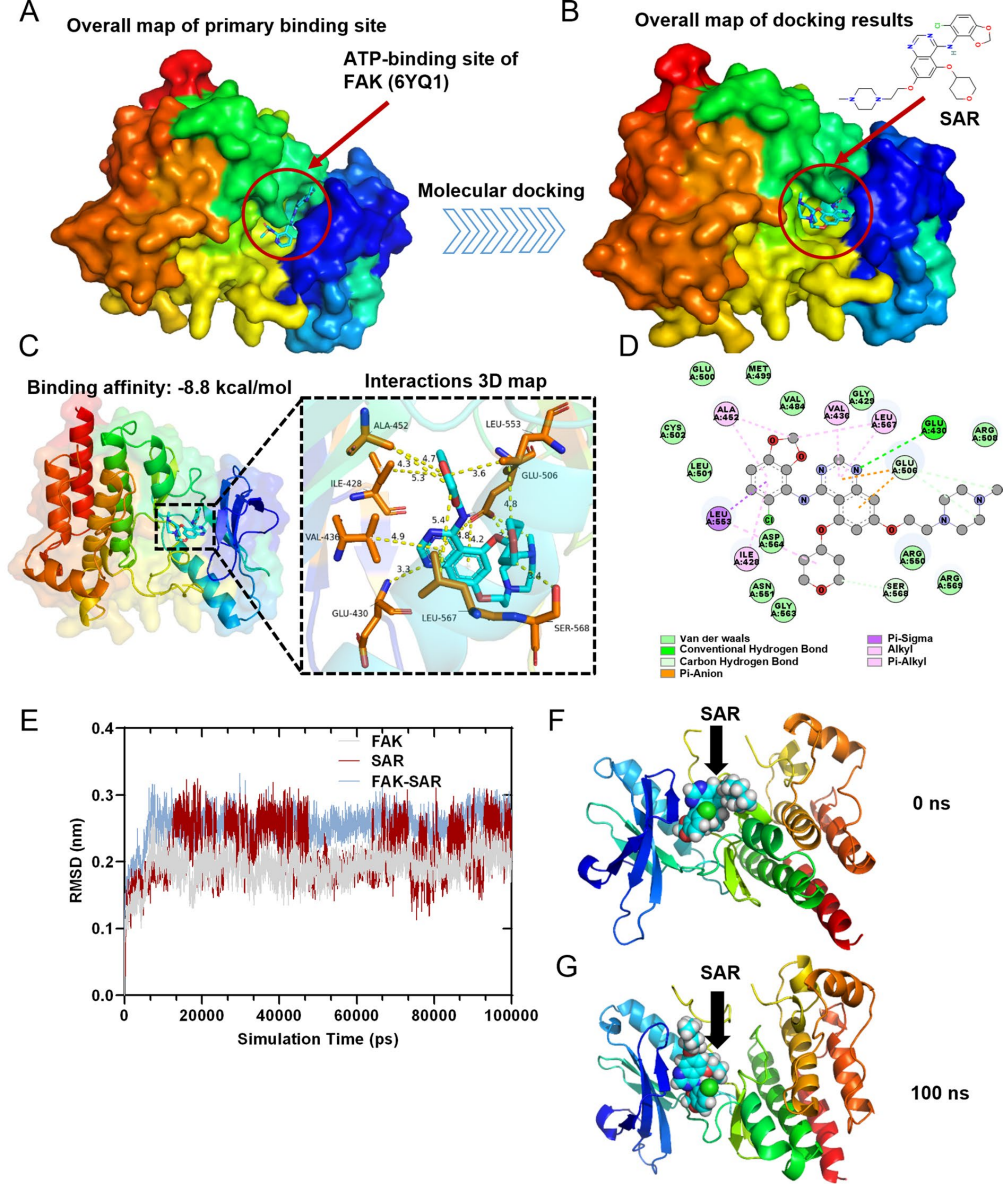
Molecular docking analysis and molecular dynamics simulation. (**A**) The crystal structure of FAK with the ATP-binding site. (**B**) Simulated docking of SAR inside the crystal structure of FAK. (**C**) Crystal structure of SAR complexed with FAK. Hydrogen bonds are shown in yellow dashed lines. The residues that can form hydrogen bonds with SAR are shown in yellow. (**D**) 2D diagram of the binding pose of SAR interacts with FAK.** (E)** RMSD Plots of RMSD of heavy atoms of FAK (grey), SAR (red), and FAK-SAR complex (blue). (**F**) Surface presentation of the FAK-SAR complex crystal structure at 0 ns. (**G**) Surface presentation of the FAK-SAR complex crystal structure at 100 ns.

Most Src inhibitors bind to its catalytic site and act as competitive ATP inhibitors, blocking the transfer of the terminal phosphate of ATP to Tyr419, whereas SAR exhibits the same inhibitory mechanism [12, 40]. To verify whether SAR can bind to FAK in a similar manner, we performed a docking study of SAR at the ATP-binding site of FAK [41] (PDB: 6YQ1) using the CB-Dock2 platform (Fig. 3A and B). The results indicated that SAR exhibited a Vina score of − 8.8 kcal/mol with FAK (Fig. 3C). Moreover, 3D and 2D interaction maps revealed that the binding of SAR to FAK was mediated by van der Waals interactions with ASP564, ARG550, VAL484, GLY429, LEU501, and other unlinked residues. SAR also exhibited stable binding to FAK through carbon and conventional hydrogen bonding with GLU430, GLU506, and SER568. The stable binding of SAR to FAK involved pi-sigma, pi-anion, pi-alkyl, and alkyl interaction bonds with residues ALA452, VAL436, LEU567, ILE428, and LEU553 (Fig. 3C and D).

To further verify the molecular docking results, the best conformation of the FAK-SAR complex was selected as the starting conformation for the molecular dynamics (MD) simulation using Gromacs2022.3 software. The root mean square deviation (RMSD) was used to examine the mobility of the receptor–ligand complex, and the RMSD curve revealed protein conformational changes. The RMSD track of the FAK mildly fluctuated around 0.2 Å over 10–100 ns (Fig. 3E). Therefore, the RMSD track of FAK indicated that it was stable at this stage (Fig. 3E, gray line). Meanwhile, the RMSD track of SAR exhibited a stable pose with fluctuation around 0.25 Å over 13–45 ns. The RMSD track of SAR further changed at 45 ns and fluctuated around 0.2 Å over 45–100 ns, which indicated that SAR shifted to a more stable pose (Fig. 3E, red line). After SAR docked with FAK, the RMSD track of the FAK–SAR complex stably fluctuated around 0.25 Å over 10–100 ns, which indicated that SAR and FAK could stably bond with one another (Fig. 3E, blue line). Through surface visualization models of FAK-SAR at 0 ns (Fig. 3F) and 100 ns (Fig. 3G), SAR was stably presented at the center of the FAK binding site throughout the MD simulation.

Root mean square fluctuation analysis of FAK revealed that amino acids 225–250, 525–560, and 575–580 exhibited significant volatility and greater residue flexibility compared with other regions (Figure S5A). The radius of gyration (Rg) value for the FAK protein remained stable throughout the entire MD simulation process (Figure S5B). Furthermore, the FAK–SAR complex showed the presence of stable hydrogen bonds that endured throughout the MD simulation, with a maximum of three hydrogen bonds formed in the FAK-SAR complex (Figure S5C). Using the solvent accessible surface area value for FAK, the FAK protein remained stable during the simulation process (Figure S5D), which suggested that the proteins were relatively stable and did not have obvious structural changes. Taken together, these results indicate that SAR regulates FAK activity through direct and stable binding.

### Overexpression of phosphorylated FAK inhibits the promoting effect of SAR on DE differentiation

**Fig. 4:**
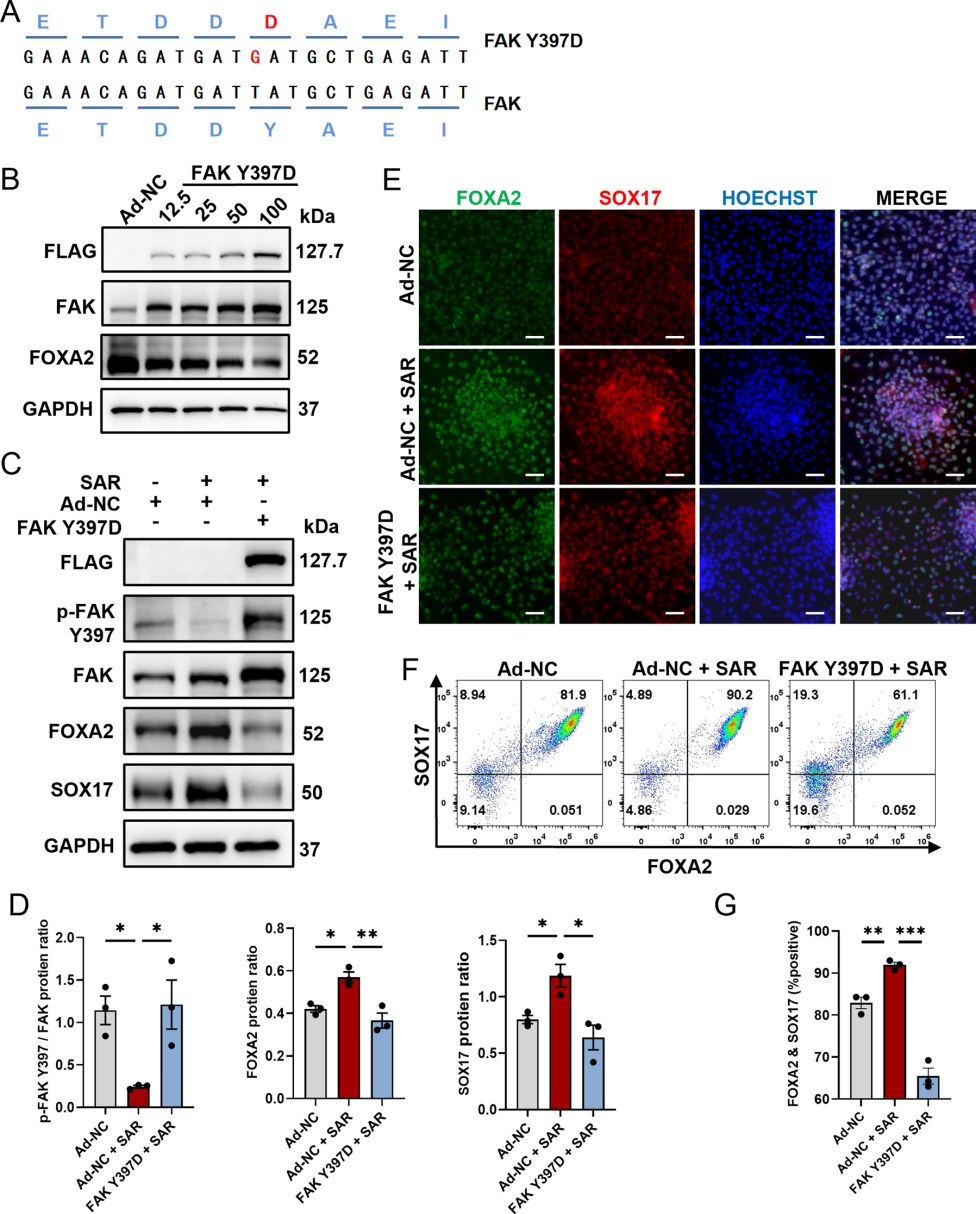
Overexpression of p-FAK diminishes the promotive effect of SAR on DE differentiation. (**A**) Sequence alignment of the FAK Y397D mutated adenoviral vector compared with wildtype FAK adenoviral vector, highlighting the Y397 pseudophosphorylation. (**B**) Human ESC line H1 was infected with various MOI of negative control adenovirus (Ad-NC) or FAK Y397D-overexpressing adenovirus during DE differentiation. Three days after differentiation, total proteins were extracted for western Blotting examination of FLAG, FAK, and FOXA2. GAPDH was used as a loading control. (**C**) Western Blotting for FLAG, p-FAK Y397, FAK, FOXA2, and SOX17 in DE cells with indicated treatments. GAPDH was used as a loading control. (**D**) Quantitative statistics of p-FAK/FAK, FOXA2, and SOX17 corresponding to (C). (**E**) Immunofluorescent staining examined FOXA2^+^ (green) and SOX17^+^ (red) cells treated with Ad-NC or FAK Y397D, in conjunction with either DMSO or SAR during 3 days of DE differentiation. The nucleus was counterstained with Hoechst 33,342. Scale bars = 100 μm. (**F**) Flow cytometric analysis of differentiated cells expressing FOXA2 and SOX17 after treatment of Ad-NC or FAK Y397D, in combination with either DMSO or SAR. (**G**) Quantitative statistics of FOXA2^+^/SOX17^+^ cells corresponding to (F). Data are presented as mean ± SEM (*n* = 3). *p < 0.05; **p < 0.01; ***p < 0.001

To further examine the role of FAK phosphorylation in DE differentiation, we used an adenovirus vector overexpressing pseudophosphorylated FAK by mutating Tyr397 into Asp397 (Fig. 4A). The optimization of the transfection conditions was done to determine the most effective parameters. The transfection efficiency reached its maximum and cell viability was most favorable at a multiplicity of infection (MOI) of 50, whereas higher MOIs resulted in significant cell death (Fig. 4B). As expected, we observed a marked upregulation of FOXA2 and SOX17 proteins following SAR treatment, whereas FAK Y397D overexpression effectively abolished the promoting effect of SAR as both SOX17 and FOXA2 were downregulated (Fig. 4C and D). The immunostaining results were consistent with those acquired by western blot analysis, which showed that FAK Y397D overexpression effectively abolished the promoting effect of SAR (Fig. 4E). Furthermore, we validated these findings by flow cytometry (Fig. 4F and G). The results indicate an important regulatory role of FAK phosphorylation in orchestrating the SAR-promoted DE differentiation process.

### Low concentrations of SAR inhibit YAP nuclear translocation and suppress FAK phosphorylation

**Fig. 5:**
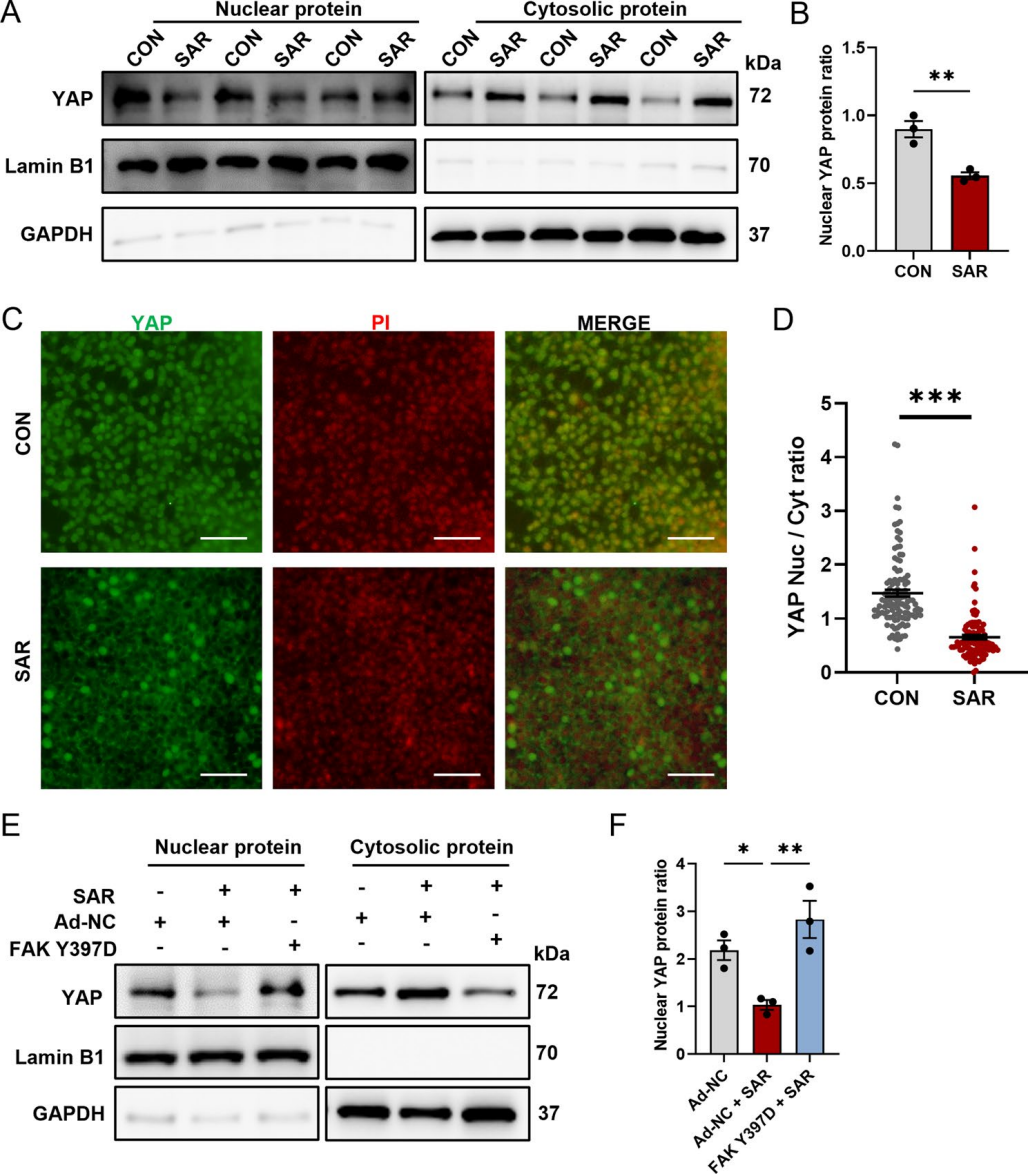
SAR inhibits YAP nuclear translocation. Human ESC line H1 was differentiated into DE cells with or without the addition of SAR. Three days after differentiation, nuclear protein and cytosolic protein were isolated separately and YAP protein level was determined by western Blotting (**A**). In the nuclear fraction, Lamin B1 was used as loading control and GAPDH as negative control; in the cytosolic fraction, GAPDH was used as loading control and Lamin B1 as negative control. (**B**) Quantitative statistics of YAP corresponding to (**A**). (**C**) Immunofluorescent staining examines the subcellular location of YAP (green) with or without 0.5 µM SAR treatment. The nucleus was counterstained with PI. Scale bars = 50 μm. The YAP fluorescent intensity within nucleus or cytoplasm was measured separately using ImageJ and the nuclear-to-cytoplasmic ratio is shown in (**D**). *N* = 118 for control group, *n* = 108 for SAR-treated group. (**E**) Western blotting for YAP in DE cells treated with negative control adenovirus (Ad-NC) or FAK Y397D-overexpressing adenovirus, in conjunction with either DMSO or SAR during 3 days of DE differentiation. In the nuclear fraction, Lamin B1 was used as loading control and GAPDH as negative control; in the cytosolic fraction, GAPDH was used as loading control and Lamin B1 as negative control. (**F**) Quantitative statistics of YAP corresponding to (**E**). Data are presented as mean ± SEM (*n* = 3). *p < 0.05; **p < 0.01; ***p < 0.001

The interplay between YAP and FAK was substantiated in numerous studies, underscoring the influence of FAK on the functional attributes and subcellular localization of YAP [31]. We examined nuclear–cytoplasmic fractionation in both control cells and those subjected to 0.5 µM SAR (Fig. 5A). The results indicated a marked and statistically significant decrease in YAP nuclear translocation (Fig. 5B). Simultaneously, an increase in the cytoplasmic content of YAP protein was observed, confirming that 0.5 μm SAR reduces YAP nuclear localization while increasing cytoplasmic localization. To confirm this observation, we performed immunofluorescence staining on DE cells with or without SAR treatment at the same cell density. The results confirmed a conspicuous reduction in YAP nuclear translocation (Fig. 5C and D). The results indicated that SAR effectively inhibited both YAP nuclear translocation and function. Conversely, overexpression of phosphorylated FAK antagonized the effects of SAR (Fig. 5E and F). These findings suggest that SAR exerts an inhibitory effect on YAP nuclear translocation by suppressing FAK phosphorylation.

### The YAP inhibitor verteporfin promotes DE differentiation by inhibiting YAP nuclear translocation

**Fig. 6:**
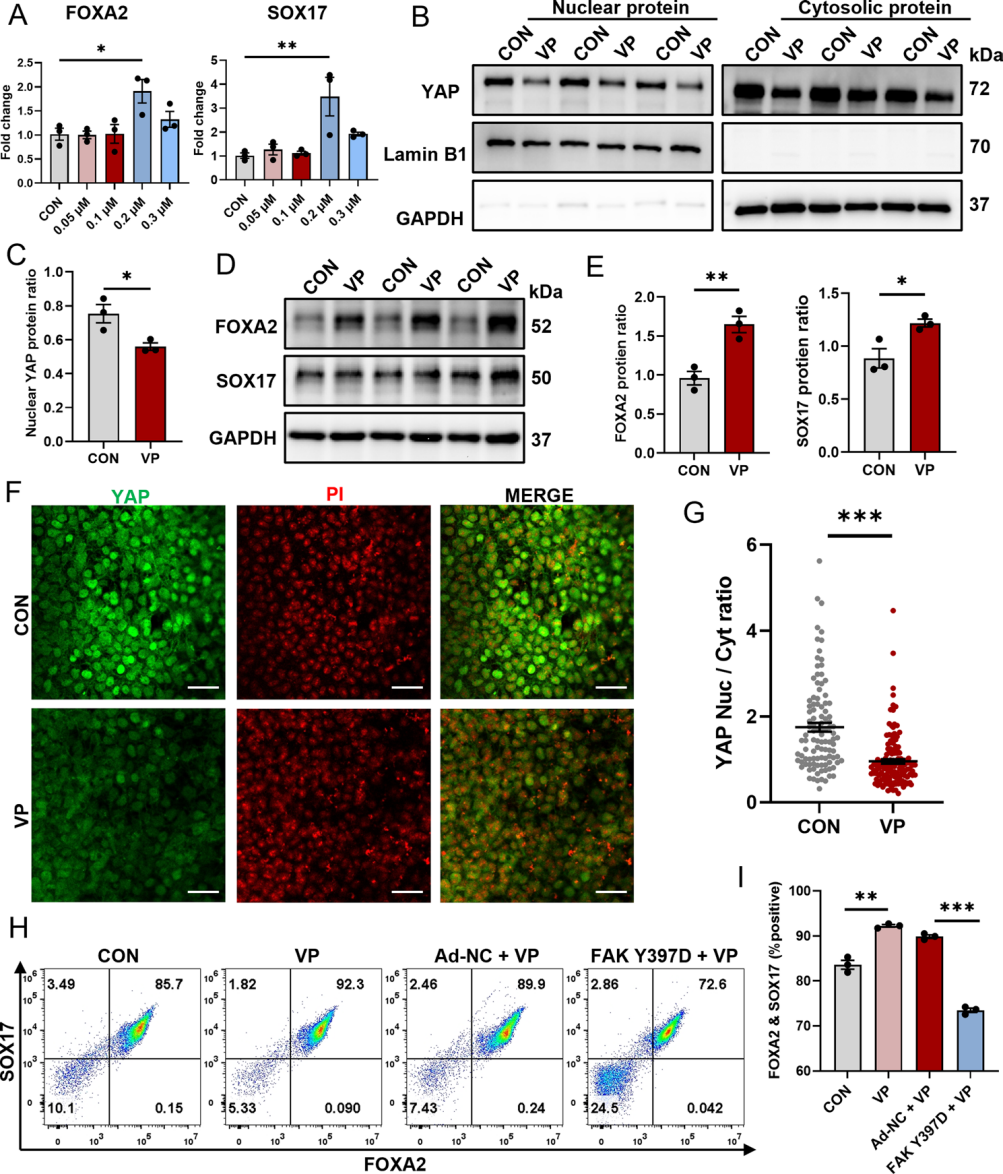
Verteporfin (VP) promotes DE differentiation by inhibiting YAP nuclear translocation. Human ESC line H1 was differentiated into DE cells with the addition of various concentrations of VP. Three days after differentiation, total RNAs were extracted and qPCR was performed to examine the expressions of FOXA2 and SOX17 **(A)**. (**B**) Nuclear protein and cytosolic protein were isolated separately and YAP protein level was determined by western Blotting. In the nuclear fraction, Lamin B1 was used as loading control and GAPDH as negative control; in the cytosolic fraction, GAPDH was used as loading control and Lamin B1 as negative control. (**C**) Quantitative statistics of YAP corresponding to (**B**). (**D**) Western blotting for FOXA2 and SOX17 in DE cells treated with 0.2 µM VP or equal volume of DMSO. GAPDH was used as a loading control. (**E**) Quantitative statistics of FOXA2 and SOX17 corresponding to (**D**). (**F**) Immunofluorescent staining of YAP (green) in DE cells with or without the treatment of 0.2 µM VP. The nucleus was counterstained with PI. Scale bars = 50 μm. The YAP fluorescent intensity within nucleus or cytoplasm was measured separately using ImageJ and the nuclear-to-cytoplasmic ratio is shown in** (G)**. *N* = 105 for control group, *n* = 137 for SAR-treated group. (**H**) Flow cytometric analysis of differentiated cells expressing FOXA2 and SOX17 with the indicated treatments. (**I**) Quantitative statistics of FOXA2^+^/SOX17^+^ cells corresponding to **(H)**. Data are presented as mean ± SEM (*n* = 3). *p < 0.05; **p < 0.01; ***p < 0.001; ns, not significant

To explore the feasibility of inhibiting DE differentiation through the direct modulation of YAP activity, verteporfin (VP), a well-documented YAP inhibitor [42], was used to elucidate the role of YAP in DE differentiation. The qPCR results indicated a dose-dependent increase in RNA levels for the key DE markers, FOXA2 and SOX17, with 0.2 µM VP without affecting cell viability (Fig. 6A). Of note, concentrations exceeding 0.2 µM adversely affected cell survival. Based on these results, 0.2 µM VP was selected as the optimal concentration for subsequent experiments. To confirm the inhibitory effect of 0.2 µM VP on YAP nuclear translocation, western blot analysis revealed that VP reduced YAP nuclear translocation (Fig. 6B and C). The results confirmed increased FOXA2 and SOX17 protein levels following the addition of 0.2 µM VP (Fig. 6D and E). To validate the direct inhibitory effect of 0.2 µM VP on YAP nuclear translocation, we performed immunofluorescence staining on DE cells with or without VP treatment at the same cell density, which revealed a marked decrease in YAP nuclear localization (Fig. 6F and G). A significant enhancement in the percentage of FOXA2^+^/SOX17^+^ cells was observed as measured by flow cytometry (Fig. 6H and I) following VP treatment. To highlight the central role of FAK as an upstream regulator of YAP and demonstrate that p-FAK overexpression counteracts the promotion of DE differentiation by VP, flow cytometry was performed. The results confirmed that FOXA2^+^/SOX17^+^ cells in the p-FAK-overexpressing group treated with 0.2 µM VP exhibited a significant reduction compared with the control group treated with the same concentration of VP (Fig. 6H and I). The results indicate the important role of the FAK/YAP axis in the regulation of DE differentiation.

### SAR reduces the requirement for AA and facilitates PSC differentiation into pancreatic progenitor cells

**Fig. 7:**
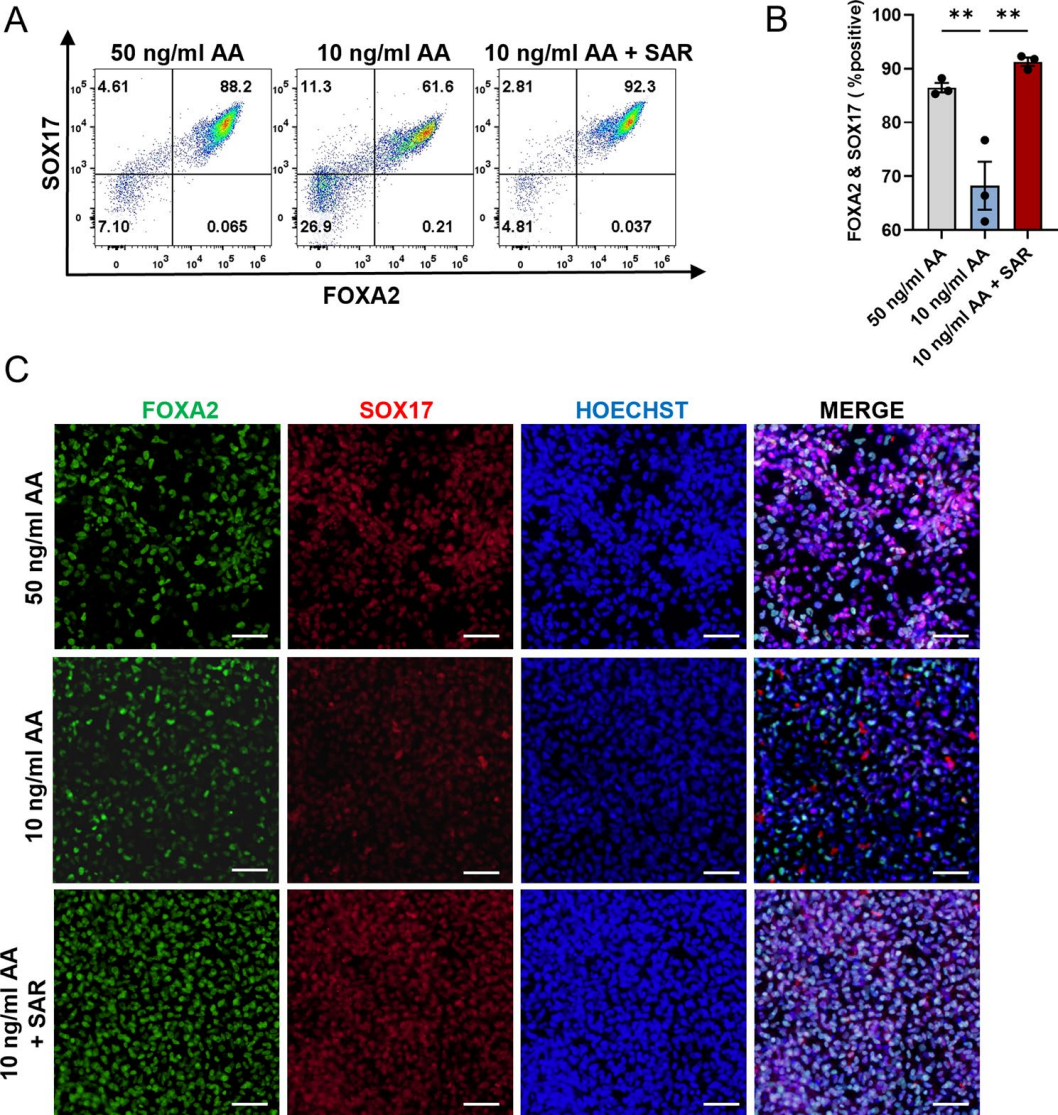
SAR reduces the requirement for AA in DE differentiation. Human ESC line H1 was differentiated into DE cells using three different conditions: 50 ng/ml AA, 10 ng/ml AA, or 10 ng/ml AA combined with 0.5 µM SAR. Three days after differentiation, cells were collected for flow cytometric analysis of FOXA2 and SOX17 (**A**). (**B**) Quantitative statistics of FOXA2^+^/SOX17^+^ cells corresponding to (A). Data are presented as mean ± SEM (*n* = 3). **p < 0.01. (**C**) Immunofluorescent staining examined FOXA2^+^ (green) and SOX17^+^ (red) cells treated with 50 ng/ml AA, 10 ng/ml AA, or 10 ng/ml AA combined with 0.5 µM SAR during differentiation. The nucleus was counterstained with Hoechst 33,342. Scale bars = 100 μm

**Fig. 8:**
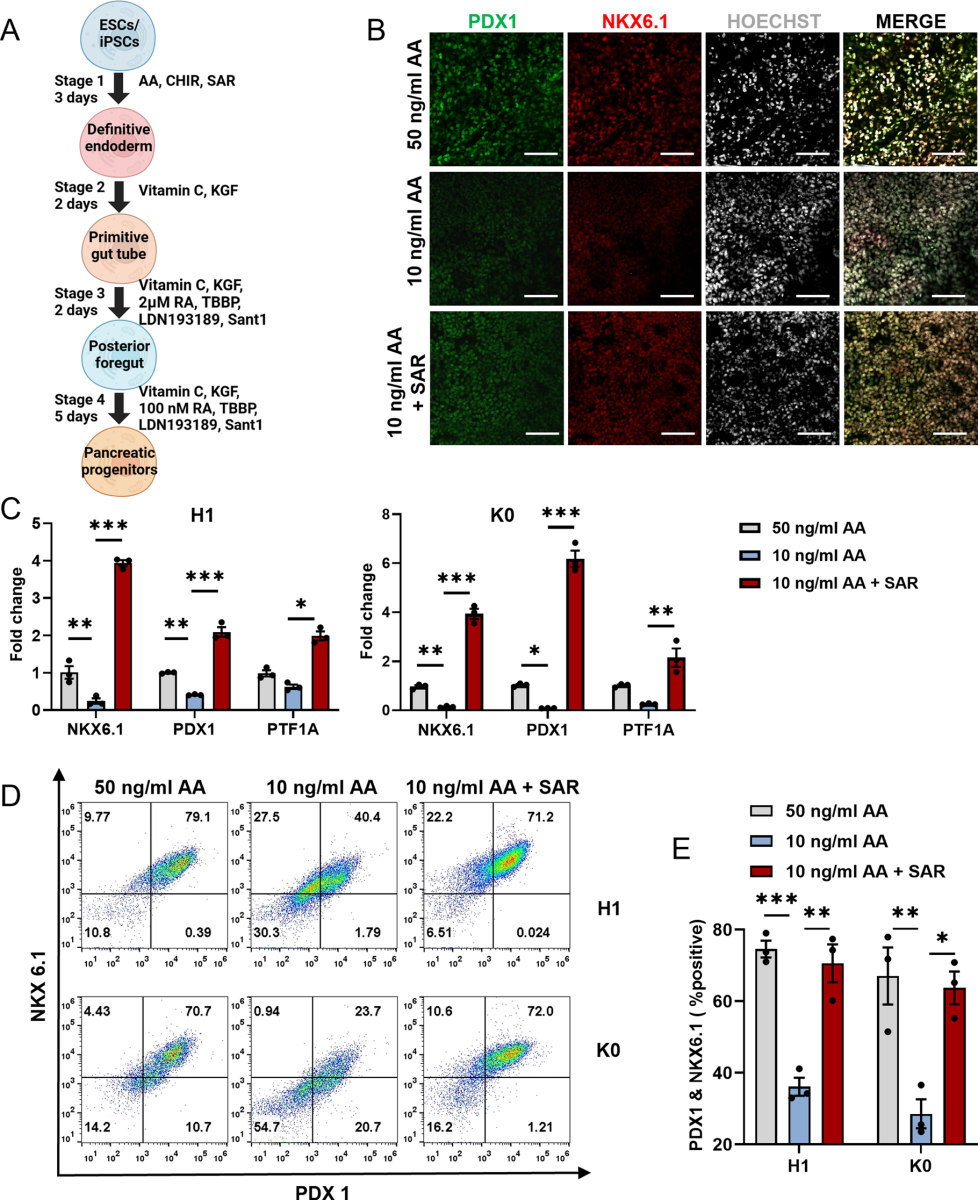
DE cells obtained from the AA-reduced differentiation protocol can further differentiate into pancreatic progenitor cells. (**A**) Schematic overview of pancreatic progenitor differentiation protocol for using SAR. (**B**) Human ESC line H1 was differentiated into DE cells using three different conditions: 50 ng/ml AA, 10 ng/ml AA, or 10 ng/ml AA combined with 0.5 µM SAR. Immunofluorescent staining was conducted to examine PDX1^+^ (green) and NKX6.1^+^ (red) cells derived from each condition during DE differentiation. The nucleus was counterstained with Hoechst 33,342. Scale bars = 100 μm. (**C**) QPCR analysis of NKX6.1, PDX1, and PTF1A expressions in pancreatic progenitor cells from human ESC line H1 and human iPSC line K0 derived from corresponding DE cells using three different conditions: 50 ng/ml AA, 10 ng/ml AA, or 10 ng/ml AA combined with 0.5 µM SAR. (**D**) Flow cytometric analysis of NKX6.1 and PDX1 in pancreatic progenitor cells differentiated from H1 and K0 cell. (**E**) Quantitative statistics of NKX6.1^+^/PDX1^+^ cells corresponding to (**D**). Data are presented as mean ± SEM (*n* = 3). *p < 0.05; **p < 0.01; ***p < 0.001

To explore the feasibility of using SAR as a partial substitute for AA to promote efficient DE differentiation, we attempted to reduce the requirement of AA in the DE differentiation process. We examined FOXA2^+^/SOX17^+^ cells after differentiation with various concentrations of AA with or without SAR using flow cytometry (Figure S6). We confirmed that reducing the concentration of AA from 50 ng/ml to 10 ng/ml decreased the proportion of FOXA2^+^/SOX17^+^ cells from 86.47% ± 0.88–68.24% ± 4.46%. Nevertheless, the introduction of 0.5 µM SAR mitigated this decline, ultimately enhancing the yield of FOXA2^+^/SOX17^+^ cells to 91.29% ± 0.77% (Fig. 7A and B). The immunofluorescence staining results also confirmed that SAR could reduce the requirement for AA during DE differentiation (Fig. 7C). To determine the competency of the DE cells for subsequent differentiation, we induced pancreatic lineage differentiation (Fig. 8A) and found that 10 ng/ml AA with the addition of SAR at the DE differentiation stage yielded a comparative number of PDX1^+^/NKX6.1^+^ pancreatic progenitor cells compared with those from 50 ng/ml AA (Fig. 8B). Using 10 ng/ml AA alone at the DE differentiation stage significantly reduced the number of pancreatic progenitor cells. The qPCR results indicated upregulation of the pancreatic progenitor markers PDX1, NKX6.1, and PTF1A in both SAR-treated human ESC line H1 and iPSC line K0, compared with the 10 ng/ml and 50 ng/ml AA groups (Fig. 8C). These data indicated that SAR-promoted DE cells at a low AA concentration were potent to differentiate into a pancreatic lineage. To further ascertain the capability of the acquired DE cells to undergo subsequent differentiation, we employed the pancreatic lineage differentiation system as previously described [35]. Our flow cytometry results revealed that 10 ng/ml AA with the addition of SAR in the DE differentiation stage yielded a comparative number of PDX1^+^/NKX6.1^+^ pancreatic progenitor cells to those from 50 ng/ml AA for DE induction (Fig. 8D and E). These results suggest that the protocol using SAR is cost-effective for DE differentiation.

## Discussion

Ensuring and enhancing the efficiency of endodermal differentiation is an important step to further improve the quality of differentiated organoids. As a nonreceptor protein-tyrosine kinase, Src has been the subject of numerous studies over three decades, in part, because of its association with malignant transformation and oncogenesis [43]. SAR has been extensively studied and used for cancer treatment; however, there have been no studies regarding its application in the field of iPSC/ESC differentiation. In the present study, we found for the first time that the Src inhibitor SAR exerts off-target effects to deactivate FAK under low concentration conditions, thereby promoting endodermal differentiation.

Numerous studies have highlighted the pivotal role of FAK in early developmental processes. For example, Afrikanova et al. demonstrated that the selective inhibition of FAK activation induces early endocrine commitment, as evidenced by an upregulation in the expression of key transcription factors, such as NGN3, NEUROD1, and NKX2.2 [44]. A parallel investigation by Liu et al. demonstrated that 3D culture conditions play an important role in promoting endocrine commitment, which is consistent with some of our results. They demonstrated that this effect is mediated by the limitation of FAK-dependent activation of the SMAD2/3 pathway. Moreover, the functional maturation of insulin-producing cells was enhanced in 3D culture, a phenomenon attributed to the upregulation of Connexin 36 expression [45]. Previous studies provide compelling evidence supporting the notion that 3D culture hampers the activation of FAK, ultimately fostering commitment to the endocrine lineage [45]. These studies support the main findings of our study. Nonetheless, we did not explore the impact of SAR on intercellular communication in the present study, which represents an intriguing avenue for future studies. While current research on stem cell differentiation into organoids has primarily focused on phenotypic analyses, mechanistic studies remain less comprehensive. Acknowledging this gap, we advocate a paradigm shift toward identifying the underlying mechanisms through which various small molecule drugs promote differentiation. Current studies have examined the downstream effects of FAK during the differentiation process. Wrighton et al. revealed that extracellular signals modulate the autophosphorylation level at the intracellular FAK 397 site through integrin signaling, thereby regulating the activation of the AKT pathway to balance the differentiation and self-renewal of embryonic stem cells [46]. Human FAK contains several tyrosine residues that can be phosphorylated, including Tyr397, Tyr407, Tyr576, Tyr577, and Tyr925 [47]. Among these, Tyr397 is the most crucial phosphorylation site, the activation of which can initiate downstream signaling. Tyr576 and Tyr577 are located within the kinase domain of FAK and serve as enhancers for the kinase activity of FAK [48]. Phosphorylation of Y576/Y577 signifies full catalytical activity of FAK [49]. Phosphorylated Y925 is a docking site for the adaptor protein Grb2 [50], which links FAK signaling to the Ras/MAPK signaling pathway. However, phosphorylated Y925 seems to have no direct relationship with the tyrosine kinase activity of FAK. In summary, we believe that the phosphorylation status of the FAK Y397 site best represents the activation or inhibition of FAK kinase activity, while Y576/577 can serve as a supportive reference, and Y925 is not strongly associated with the kinase activity of FAK. Therefore, our focus is primarily on the phosphorylation status of the Y397 site. Incorporating these insights into our understanding of FAK-mediated pathways enhances the foundation for future studies aimed at manipulating stem cell fate with precision and efficacy.

YAP, a key player in the Hippo pathway, is pivotal in directing stem cell differentiation. In the present study, we revealed the role of direct modulation of FAK397 phosphorylation in modulating the nuclear localization of YAP, which in turn, affects the efficiency of endodermal differentiation. However, while our findings shed light on this regulatory mechanism, an understanding of the precise influence of YAP on endodermal differentiation mechanisms remains unclear. Further in-depth studies are warranted to unravel the intricate role of YAP in this process. With respect to the specific regulatory role of YAP in endodermal differentiation, our hypothesis revolves around its potential to suppress the pluripotency of ESCs. Qin et al. demonstrated that YAP overexpression drives a naive state in human PSCs and is indispensable for the efficient self-renewal of human naive PSCs [51]. The Hippo pathway governs organ size, regeneration, and cell growth by modulating the stability of transcription factors YAP1 and TAZ. Activation of the Hippo/MST kinase cascade results in the phosphorylation of YAP1/TAZ, leading to their degradation. In instances where Hippo kinase is inactive, YAP1/TAZ translocate to the nucleus, where they bind to TEAD family members 1–4 DNA-binding proteins and regulate transcription [52]. Moreover, findings from several research groups suggest that YAP1 impedes the differentiation of mesendoderm (ME) in hESCs by influencing the activity of the Nodal and WNT3 pathways [53, 54]. This evidence underscores the significant role of YAP1 in primitive gut formation, although further functional investigations are necessary to elucidate its precise involvement in this developmental process. Conchi Estarás et al. have discovered that the Hippo pathway regulator, YAP, cooperates with TEAD to modulate the binding of the negative elongation factor and suppress SMAD2,3 induction of ME genes. Consequently, the Wnt3a/β-catenin and Activin/SMAD2,3 pathways synergize to counteract YAP repression and amplify ME gene expression during the early differentiation of hESC [55]. Furthermore, numerous studies indicate that functional YAP plays a significant role in regulating the self-renewal of embryonic stem cells. Christoffer Tamm et al. demonstrate that YAP increases the activity of the Oct-3/4 and Nanog promoters, where Oct4 and Nanog serve as crucial pluripotency markers [56]. In addition to suppressing stem cell pluripotency, it is speculated that the promotion of DE differentiation upon nuclear YAP reduction may also be due to enhanced differentiation of the anterior primitive streak (APS), thereby facilitating endoderm differentiation. Originating from the APS, the endoderm is inhibited by YAP, as demonstrated by Hui-Ting Hsu and colleagues’ study. Brief exposure of hESCs to dasatinib, an effective YAP inhibitor, leads to differentiation into APS-derived endoderm and mesoderm in response to AA [54]. Consequently, there is still considerable research needed to delve into the precise mechanisms by which YAP influences DE differentiation.

In addition to YAP, SAR may have other potential mechanisms for promoting DE differentiation that have not been investigated in this study. For example, Akt was reported as a downstream target of FAK that involved in the regulation of DE differentiation [57]. McLean et al. [58]revealed that suppression of Akt is indispensable during AA-induced DE differentiation. Consistently, a recent study showed that inhibition of PI3K/Akt signaling improved the exit from pluripotency and DE differentiation [59]. Further investigation is warranted to comprehensively understand the intricate regulatory network orchestrated by SAR during DE differentiation.

During our flow cytometry analysis to validate the functionality of 0.5µM SAR, we observed a dim subpopulation for FOXA2 present in the double-positive quadrant. To the best of our knowledge, we believe this FOXA2^low^ population represents an early stage of DE differentiation. This population was also identified by previous study, but did not define the function of these cell [60]. We hypothesize that SAR not only enhanced the population of FOXA2/SOX17 double-positive cells but also increased the FOXA2 expression in the FOXA2^low^ population, resulting in an increase of total FOXA2^+^ population. To gain deeper insights into this phenomenon, we are considering employing single-cell RNA-sequencing to elucidate how these FOXA2^low^ cells were regulated by SAR.

## Conclusion

In conclusion, our present study demonstrated that low concentrations of SAR promote DE differentiation by inhibiting the FAK/YAP axis. This mechanism holds promise for optimizing DE differentiation protocols and potentially reducing the requirement for AA. Our findings highlight the significance of FAK in developmental biology and regenerative medicine, shedding new light on the intricate mechanisms underlying cell fate determination.

### Electronic supplementary material

Below is the link to the electronic supplementary material.

**Supplementary Material 1:** Supplementary Table 1

**Supplementary Material 2:** Supplementary Table 2

**Supplementary Material 3:** Supplementary Figure 1

**Supplementary Material 4:** Supplementary Figure 2

**Supplementary Material 5:** Supplementary Figure 3

**Supplementary Material 6:** Supplementary Figure 4

**Supplementary Material 7:** Supplementary Figure 5

**Supplementary Material 8:** Supplementary Figure 6

## Data Availability

The RNA-seq data were deposited in the GEO DataSets under submission number GSE251992. All presented data in this study are available from the corresponding author upon reasonable request.

## Abbreviations

DE: Definitive endoderm
SAR: Saracatinib
FAK: Focal adhesion kinase
YAP: Yes-associated protein
PSCs: Pluripotent stem cells
ESCs: Embryonic stem cells
iPSCs: induced pluripotent stem cells
AA: Activin A
TEAD: TEA domain transcription factor
RNA-seq: RNA sequencing
PCA: Principal component analysis
GO: Gene ontology
MD: Molecular dynamics
RMSD: Root mean square deviation
RMSF: Root mean square fluctuation
Rg: Radius of gyration
SASA: Solvent accessible surface area
MOI: Multiplicity of infection
VP: Verteporfin
ME: Mesendoderm
NELF: Negative Elongation Factor
APS: Anterior Primitive Streak
