# Supplementary figures and images for: Low concentrations of saracatinib promote definitive endoderm differentiation through inhibition of FAK-YAP signaling axis
Source: Cell Commun Signal. 2024 May 30;22:300. doi: 10.1186/s12964-024-01679-7 (PMC11140888; doi:10.1186/s12964-024-01679-7)

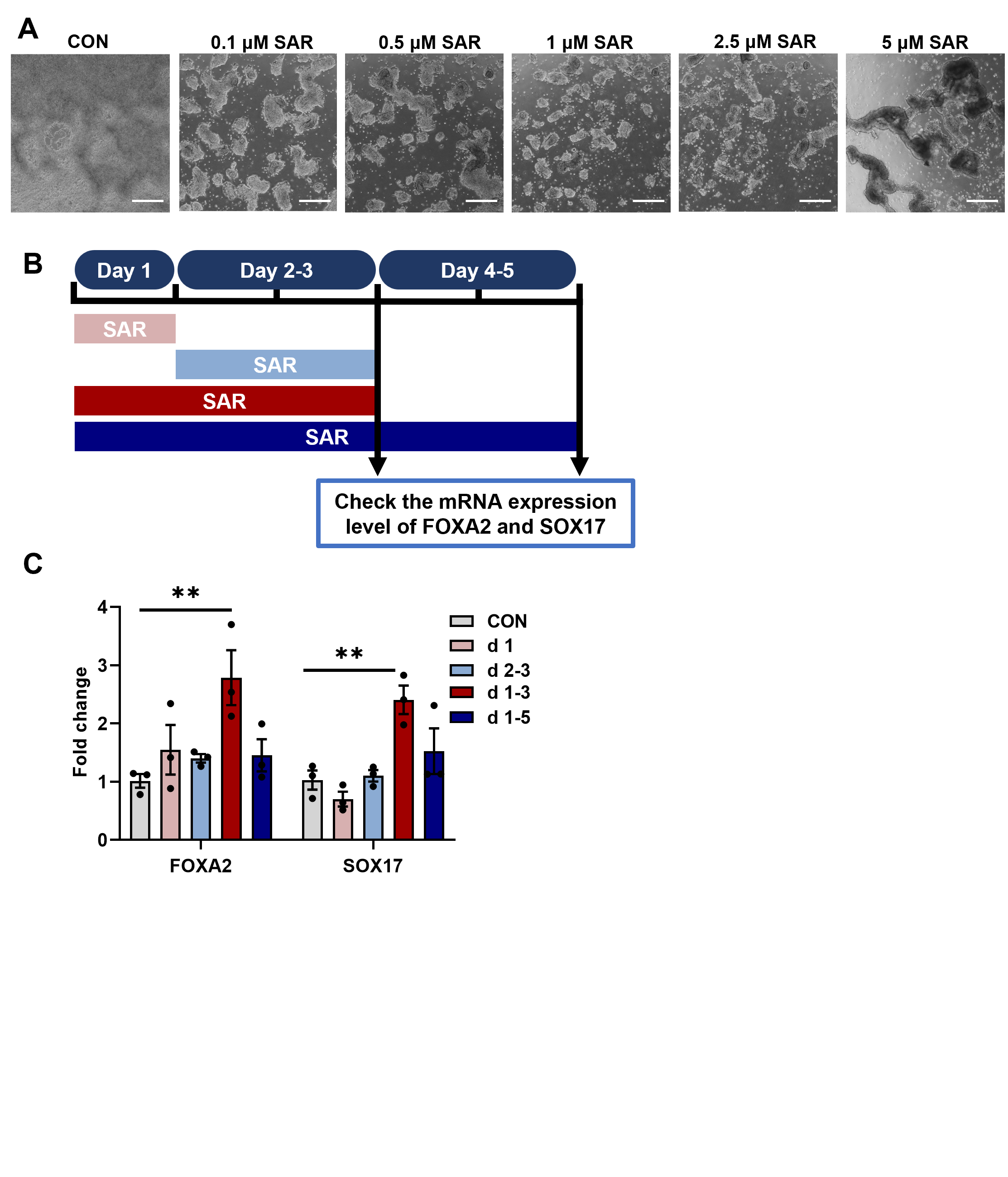

Supplement: Supplementary file 3 — Supplementary Material 3: Supplementary Figure 1 [file 12964_2024_1679_MOESM3_ESM.tif]

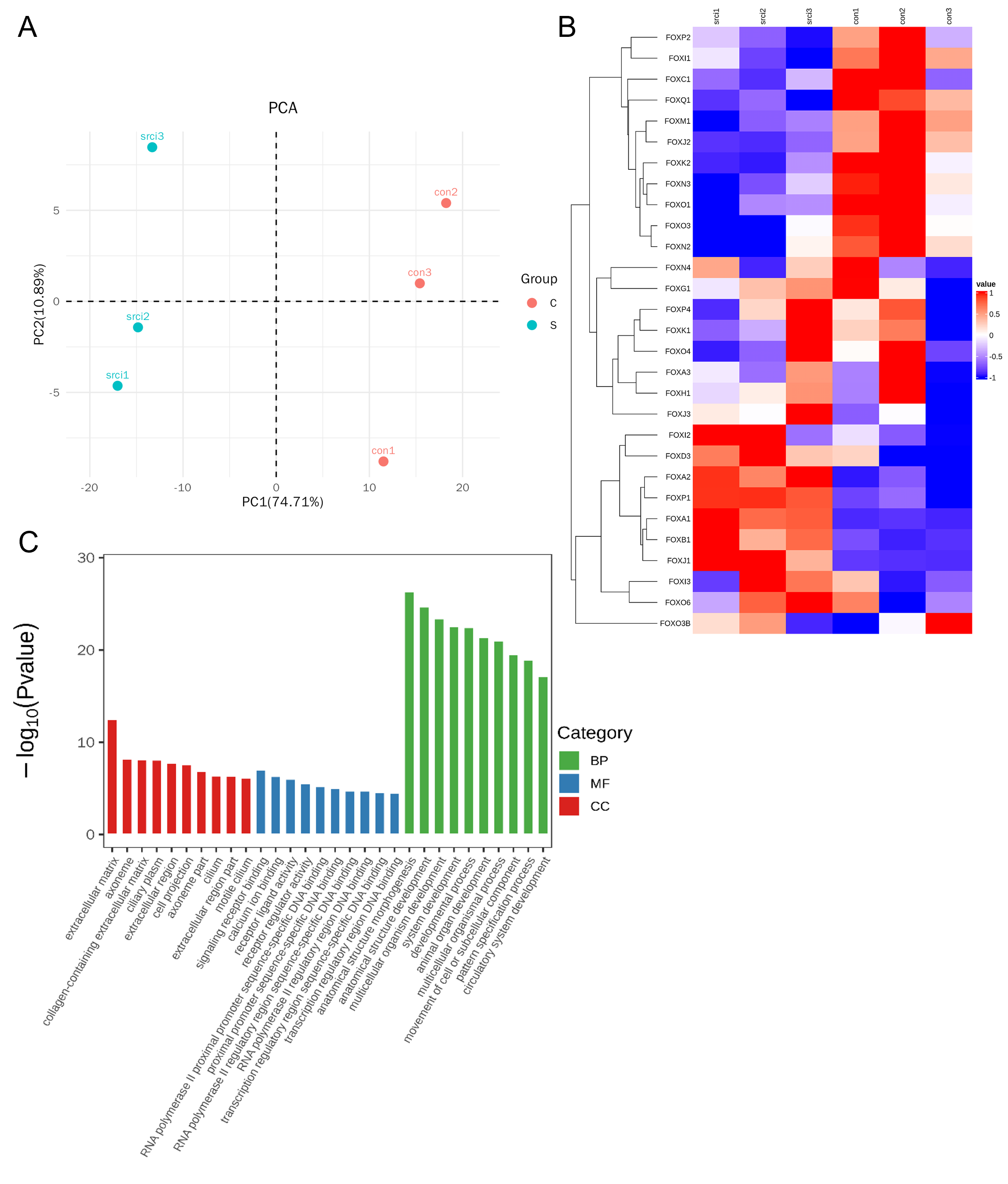

Supplement: Supplementary file 4 — Supplementary Material 4: Supplementary Figure 2 [file 12964_2024_1679_MOESM4_ESM.tif]

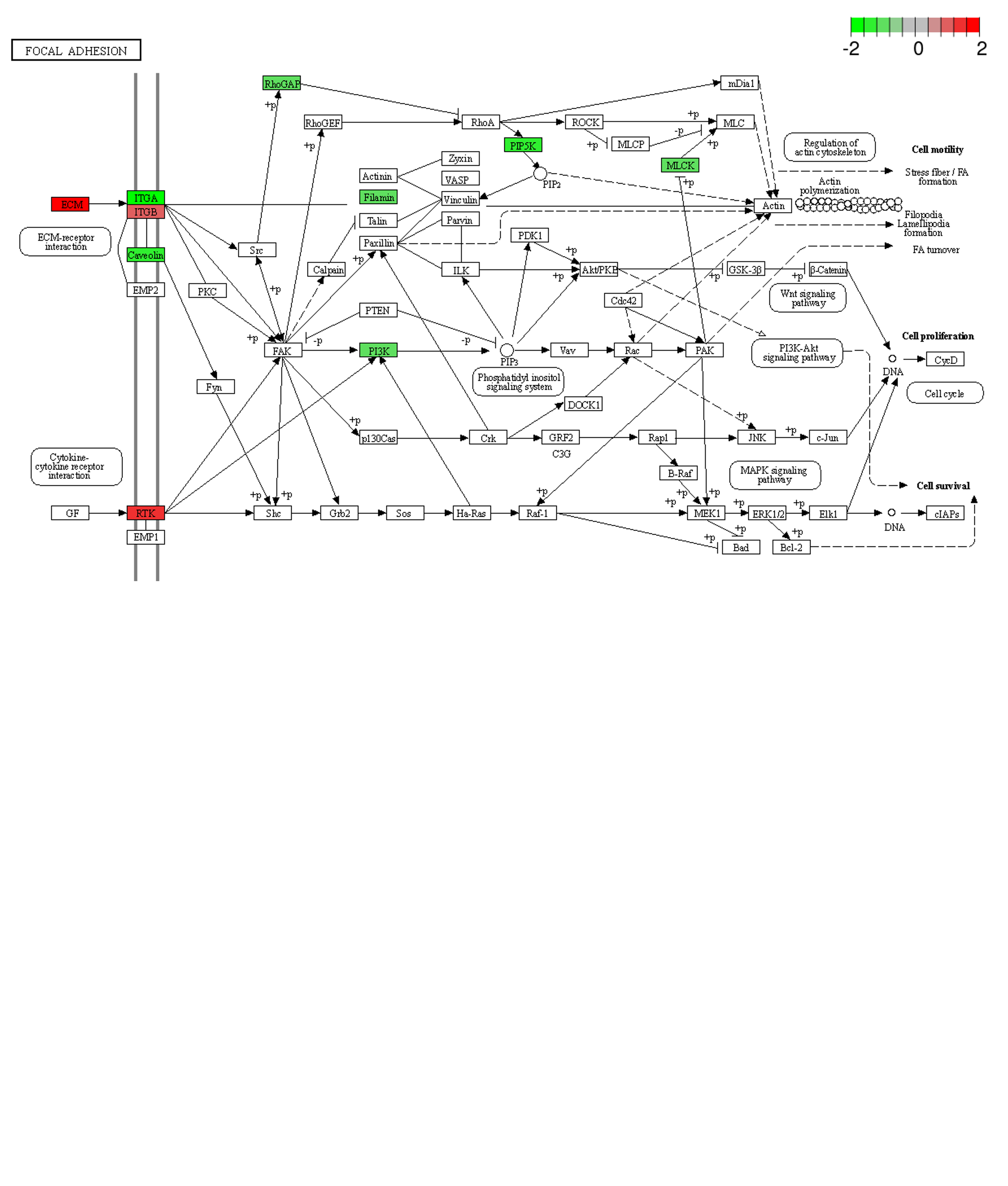

Supplement: Supplementary file 5 — Supplementary Material 5: Supplementary Figure 3 [file 12964_2024_1679_MOESM5_ESM.tif]

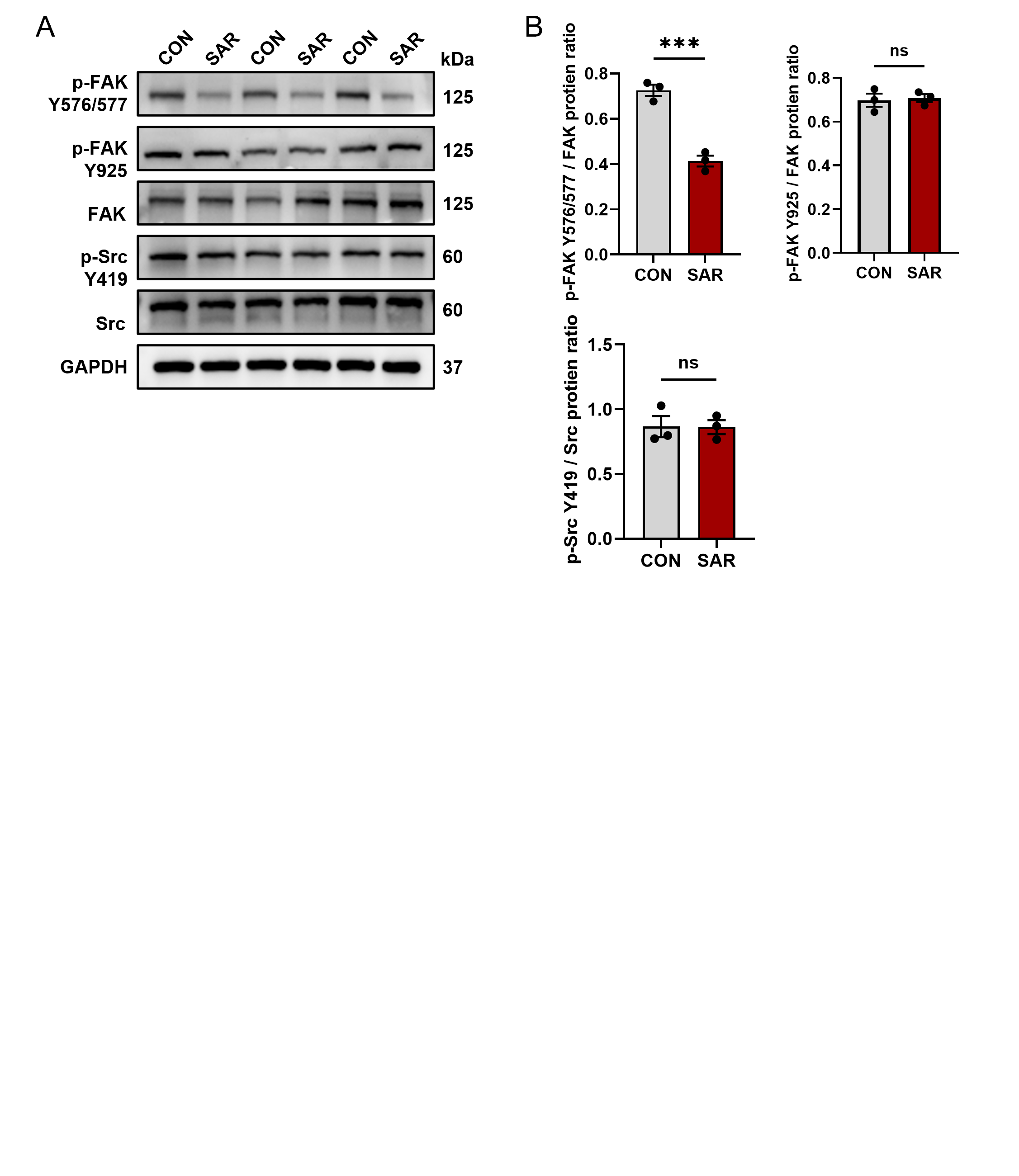

Supplement: Supplementary file 6 — Supplementary Material 6: Supplementary Figure 4 [file 12964_2024_1679_MOESM6_ESM.tif]

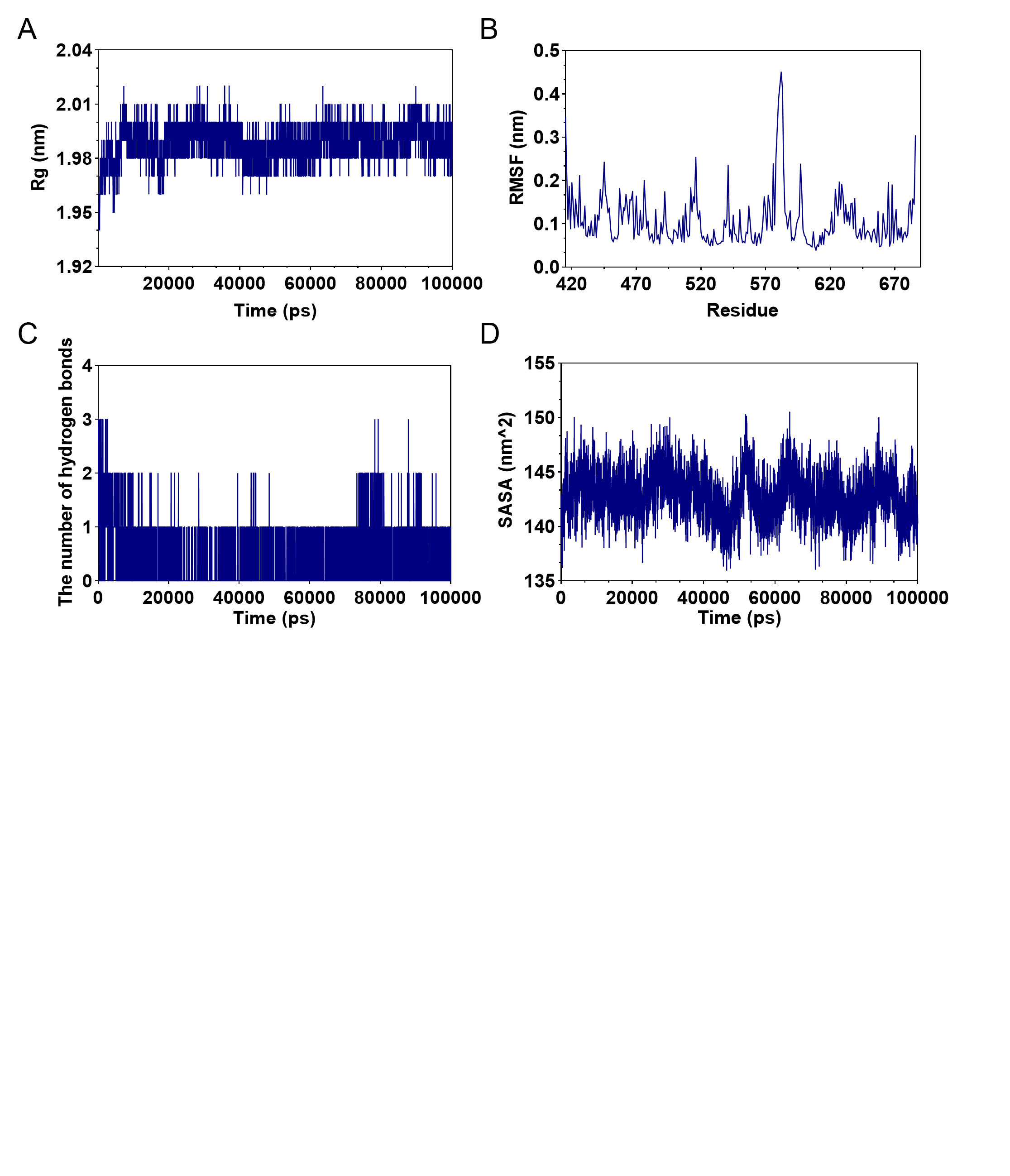

Supplement: Supplementary file 7 — Supplementary Material 7: Supplementary Figure 5 [file 12964_2024_1679_MOESM7_ESM.tif]

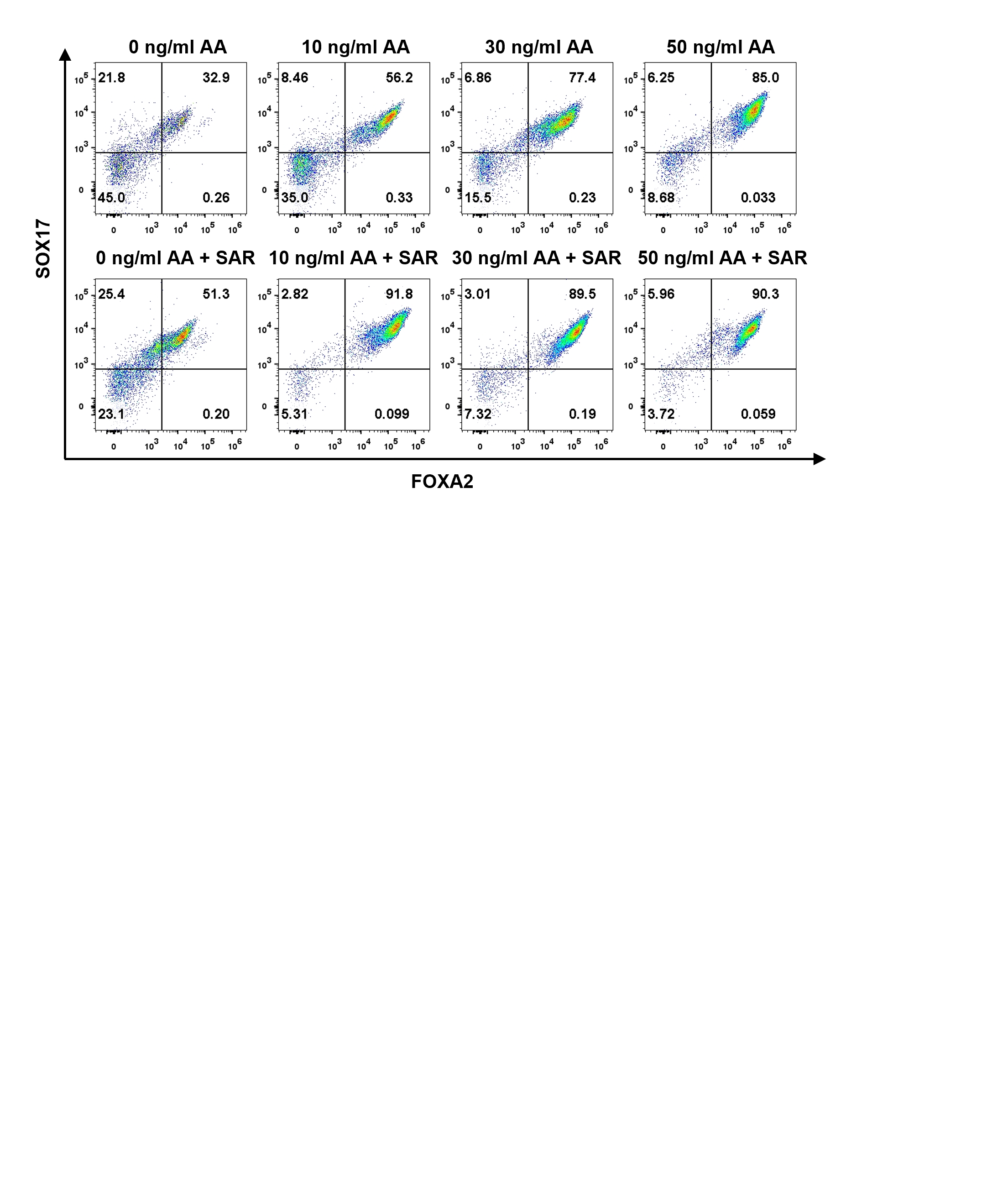

Supplement: Supplementary file 8 — Supplementary Material 8: Supplementary Figure 6 [file 12964_2024_1679_MOESM8_ESM.tif]
